# Impact of Post‐COVID Syndrome on Cardiorespiratory Fitness, Psychological Well‐Being, and Quality of Life in Adolescents: A Cross‐Sectional Study

**DOI:** 10.1155/pm/5599011

**Published:** 2026-03-11

**Authors:** Albane Bertha Rosa Maggio, Ivan Perret, Nuseibah Alramadina, Anne Perrin, Constance Barazzone, Anne Mornand

**Affiliations:** ^1^ Department of Pediatrics, Gynecology and Obstetrics, University Hospitals of Geneva, Geneva, Switzerland, hug-ge.ch; ^2^ Faculty of Medicine, University of Geneva, Geneva, Switzerland, unige.ch

**Keywords:** breathing pattern, cardiorespiratory exercise test, cardiorespiratory fitness, dysfunctional breathing, peak oxygen consumption, post- syndrome, pulmonary function

## Abstract

**Background:**

Post‐COVID syndrome (PCS) in adolescents, marked by persistent symptoms such as dyspnea and fatigue, remains poorly understood, particularly in those referred for exercise intolerance.

**Objective:**

The objective of this study is to describe the clinical presentation and cardiorespiratory fitness (CRF) of adolescents with PCS and identify factors distinguishing those with normal versus reduced CRF.

**Study Design:**

In this cross‐sectional study, 31 adolescents (90% female) with PCS underwent cardiopulmonary exercise testing (CPET), pulmonary function tests, and completed validated questionnaires assessing fatigue, depression, hyperventilation, physical activity, and quality of life (QoL). Patients were grouped by CRF status and compared.

**Results:**

Symptoms were more prevalent than in general PCS literature, likely due to referral bias. Moderate depression risk was present in 35%, and 75% reported QoL impairment comparable with chronic conditions. Nearly half (48%) had reduced CRF. CRF was not associated with acute infection severity but correlated with orthostatism, reduced O₂ pulse, and increased static air trapping (*p* < 0.05). Preinfection physical activity was positively associated with CRF (*p* = 0.014), whereas postinfection activity levels were similar across groups.

**Conclusion:**

PCS significantly impacts CRF, QoL, and psychological well‐being in adolescents with exercise intolerance. Reduced CRF appears multifactorial, involving autonomic dysfunction, pulmonary limitations, and deconditioning. These findings underscore the need for comprehensive evaluation and targeted management strategies in this vulnerable population.

## 1. Introduction

The prevalence of post‐COVID syndrome (PCS) in children and adolescents after a SARS‐CoV‐2 infection ranges between 5% and 33% [1, 2]. PCS symptoms are highly diverse [3], but up to 20% of patients report exercise intolerance [1]. Cardiopulmonary exercise test (CPET) is the test of choice to differentiate various causes of dyspnea and exercise intolerance [4], as well as to assess cardiorespiratory fitness (CRF). A recent meta‐analysis demonstrated that exercise capacity was reduced by almost 5 mL/kg/min among adults with PCS compared with individuals without symptoms, 3 months after the acute infection [5]. This difference may be even greater when compared with a control group and has been shown to be associated with ventilatory inefficiency and impaired systemic oxygen extraction [6]. The meta‐analysis concluded that exertional intolerance may be multifactorial, involving deconditioning (mainly in individuals hospitalized for severe acute COVID‐19), dysfunctional breathing, chronotropic incompetence, and abnormal peripheral oxygen extraction [5]. Nevertheless, the majority of authors did not stratify patients according to their CRF levels, which might provide important insights into the factors underlying CRF limitations. Furthermore, to our knowledge, only one study has performed CPET in children and adolescents with PCS [7].

The primary aim of this study was to describe the clinical presentation of children and adolescents with PCS addressed for CRF evaluation. The second goal was to determine whether PCS patients with abnormal CRF share common characteristics in questionnaire results, CPET, and pulmonary function test (PFT) that allow them to be distinguished from PCS patients with normal CRF. Finally, we examined whether there was any correlation between symptomatology and physical and psychological findings.

## 2. Material and Methods

### 2.1. Study Design and Subjects

This is a cross‐sectional study held in a tertiary care center between May 2021 and February 2023. We recruited children and adolescents with PCS referred by the specialized COVID consultation for an evaluation of exercise‐induced dyspnea. We include all participants who underwent both CPET and PFT.

PCS was defined according to the WHO Delphi consensus as symptoms occurring 3 months after the onset of COVID‐19, lasting for at least 2 months, and not attributable to an alternative diagnosis [8].

The cantonal ethics committee (CCER) approved the study (CCER 2020‐00414). Written informed consent was obtained from the adolescent or their parents.

Patient and public involvement: Patients and public has not been involved in the design, conduct, reporting or dissemination plans of the research.

### 2.2. Measures

#### 2.2.1. Anthropometrics

We assessed body weight (kg) in light clothing (underwear and T‐shirt) and height (cm) without shoes. Body mass index (BMI) was calculated as weight/height squared (kg/m^2^), and *z*‐scores (zs) were derived using World Health Organization references [9].

#### 2.2.2. Questionnaires

##### 2.2.2.1. Physical Activity (PA) Questionnaires.

We used the French adaptation of the Youth Risk Behavior Surveillance System (YRBSS) questionnaire for children aged 11–14 years. This questionnaire assessed the participants′ PA levels over the previous week. We recorded the number of days the adolescent engaged in: (1) at least 20 min of intense PA (J1), (2) at least 30 min of moderate PA (J2), and (3) at least 30 min of school PA (Js). Based on their responses, we calculated the minimum amount of PA performed per week as follows: (2.5 × J1 × 20 min) + (J2 × 30 min) + (Js × 30 min). The YRBSS questionnaire is considered reliable and valid in the age 14–18 years old [10].

In addition to this questionnaire, we asked about the number of hours they spent in organized physical activities before the COVID infection and at the time of the evaluation.

##### 2.2.2.2. Quality of Life (QoL) Questionnaire—PedsQL 4.0‐SF‐15 [11].

This survey consists of 15 questions categorized into four functional domains: physical, emotional, social, and school. Each domain includes a specific number of questions: five for physical, four for emotional, three for social, and three for school. Participants responded to these questions using a Likert scale ranging from 0 (*Never*) to 4 (*Almost always*). Following the provided instructions, we calculated functioning scores for each domain by summing up the responses. Global QoL score was calculated for each participant, ranging from 0 to 100, with higher scores indicating a better QoL. Cutoffs have been proposed to distinguish between light, moderate, or major chronic conditions in children over 8 years old, at scores 78, 76, and 70, respectively [12].

##### 2.2.2.3. Nijmegen Questionnaire [13].

The Nijmegen questionnaire provides a comprehensive view of symptoms associated with hyperventilation syndrome, a type of dysfunctional breathing, demonstrating excellent sensitivity (91%) and specificity (95%) [13]. Participants evaluated the frequency of 15 symptoms on a Likert scale ranging from 0 (*Never*) to 4 (*Very often*). These symptoms include chest pain, blurred vision, dizziness, confusion, fast or deep breathing, shortness of breath, tightness across the chest, a bloated sensation in the stomach, tingling in fingers and hands, difficulty breathing or taking deep breaths, stiffness or cramps in fingers and hands, tightness around the mouth, cold hands or feet, palpitations in the chest, and anxiety. A total score over 20 indicates significant hyperventilation, whereas a score between 10 and 20 indicates mild hyperventilation.

##### 2.2.2.4. Adolescent Depression Rating Scale (ADRS).

The ADRS is a 10‐item self‐report scale validated for assessing the risk of depression in adolescents [14]. Adolescents respond by true/false to 10 statements. A total of 4 to 7 “True” responses indicates a moderate risk of depression, whereas a score of 8 or more suggests a high risk of depression.

##### 2.2.2.5. Fatigue Severity Scale (FSS).

The FSS is a 9‐item questionnaire using a Likert scale from 1 (*Strongly disagree*) to 7 (*Strongly agree*). The total score is divided by 9 to calculate a mean score, with higher scores indicating greater fatigue severity. A mean score above 4 has been validated as the cutoff for clinically significant fatigue in a large cohort [15].

#### 2.2.3. PFT

All subjects underwent spirometry using a cabin plethysmograph (BodyBox 5500, Medisoft, Leeds, United Kingdom) following the 2005 American Thoracic Society/European Respiratory Society (ATS/ERS) standards. Spirometry was considered interpretable if it met the ATS/ERS criteria [16]. We measured mechanics, volumes, and diffusion parameters. Prediction equations from the Global Lung Function Initiative (GLI 2012) [17] were used to calculate zs and percent‐predicted values. The GLI 2012 predicted values are validated for multiethnic children [18] and Caucasian Australasians [19]. The ATS and ERS recommend an age‐specific lower limit of normal (LLN) set at the 5th percentile, corresponding to a zs of −1.64 [20–22]. Unlike percent‐predicted values, zs are unbiased with respect to age, height, sex, and ethnicity.

According to the latest 2022 ERS/ATS interpretative recommendations [20], airflow obstruction was defined as an forced expiratory volume in 1 s (FEV_1_)/forced vital capacity (FVC) ratio zs < −1.64, whereas restrictive airflow was defined by FEV1, FVC, and TLC zs < −1.64 with a normal FEV_1_/FVC ratio zs. Gas transfer impairment was defined as a D_LCO_zs < −1.64 (D_LCO_ = K_CO_ x V_A_). Static air trapping or hyperinflation was indicated if the RV/TLC predicted ratio zs was above the 95th percentile, and dynamic air trapping was defined as an FVC/VC ratio (L) < 90% [23]. Bronchodilator responsiveness (BDR) was defined as the percentage change in FEV_1_ or FVC relative to the individual′s predicted values, with a change > 10% indicating a positive response.

#### 2.2.4. Cardiopulmonary Exercise Test (CPET)

Participants were instructed to refrain from any PA for at least 24 h prior to the scheduled test. They were also advised not to consume a large meal within 2 h before the test and to wear comfortable clothing. CPET was performed on a pediatric cycle ergometer, with oxygen consumption measured by direct gas analysis (MetaLyzer 3B/II, Cortex, Leipzig, Germany). The MetaLyzer 3B operates as a breath‐by‐breath device, whereas MetaLyzer II uses a mixing chamber technique. Full calibration was performed before each test. Data were analyzed using ABRM on the manufacturer′s software (MetaSoft, Cortex, Leipzig, Germany). For all subjects, blood gasses were measured from capillary blood samples taken from the fingertip before and after the test. Hemoglobin level, pH, partial pressure of oxygen (PaO_2_), and lactate were also recorded.

Prior to the test, spirometry was conducted to measure FEV1, FVC, and the FEV1/FVC ratio (FEV1/FVC%). Results were expressed as zs according to spirometry reference equations, with a zs < −1.64 considered abnormal [20].

Subjects followed a ramp protocol, consisting of baseline measurements at rest for 1 mi; warm‐up phase (10, 20, or 25 W) for 3 min; incremental exercise with fixed increments of 10, 15, 20, or 25 W per minute at a pedaling rate of 60–80 revolutions per minute; a 3‐min recovery phase at 20 W; and 2 min without pedaling.

The test was considered maximal if at least three of the following criteria were met: a score of 8/10 on the Borg scale; achievement of a VO_2_ plateau; respiratory exchange ratio (RER) > 1.05; peak heart rate (HR) > 90*%* of predicted; breathing reserve (BR) < 20%; pH < 7.32 or a drop of at least 0.04; and lactate level > 6 mmol/L [24–26].

Variables were recorded at peak and at the first ventilatory threshold for VO_2_. A VO_2_ below 40% of predicted maximal VO_2_ at VT1 was considered abnormal. O_2_pulse and maximal work zs were calculated [27]. CRF level was assessed based on the zs of peak oxygen consumption (peakVO_2_) [28], with a zs < −1.64 considered abnormal for all variables.

Exercise‐associated dysautonomia was diagnosed if all the three Jouven′s criteria were met [29]: (1) resting HR >75 bpm; (2) a difference between peak HR and resting HR < 89 bpm; (3) HR recovery in the first minute < 25 bpm.

Subjects were categorized into two groups based on their CRF level [27]: (1) normal CRF, and (2) low CRF (peakVO_2_zs < –1.64).

### 2.3. Sample Size

The sample size was not predetermined; therefore, we calculated the statistical power of our study based on the number of participants who met the inclusion criteria during the study period. With 13 subjects in the low CRF group and 18 in the normal CRF group, the power was estimated at 0.755 for detecting a difference in peak VO₂ of 6 mL/kg/min, assuming a standard deviation of 6.

### 2.4. Statistical Analysis

Statistical analyses were performed using SPSS software 25.0 (Chicago, Illinois). Data were screened initially for normality with the Kolmogorov–Smirnov test. Descriptive statistics included frequency distributions for categorical variables and means with standard deviations (SD) for continuous variables with normal distribution. There was no missing data for CRF and PFT outcomes. If other data were missing, the subjects was excluded from the specific analysis.

Statistical differences between subject categories were analyzed using Welch′s *t*‐test for continuous variables and chi‐square tests for categorical variables, as appropriate. The association between variables was evaluated using Spearman′s correlation coefficient. Differences were considered statistically significant at *p* < 0.05.

## 3. Results

### 3.1. Patient Characteristics

Thirty‐one subjects were referred for CPET evaluation, comprising three boys and 28 girls aged between 11 and 19 years (mean age 14.8 (±2.0) years). Patient characteristics and anthropometric data are presented in Table 1. All participants were of Caucasian origin. Of these subjects, 93.5% had no known respiratory diseases prior to COVID‐19 infection. Only two had mild intermittent asthma, though neither was under medication at the time of evaluation.

**Table 1:** Subjects′ characteristics and questionnaires results.

	Whole cohort *N* = 31	Normal CRF *N* = 18	Low CRF *N* = 13	
	**N (%) or mean (SD)**			**p**
Female *n*, (%)	28 (90.3%)	17 (94.4%)	11 (84.6%)	0.361

Age (years)	14.8 (2.0)	14.6 (2.4)	15.1 (1.4)	0.501

*Anthropometrics* (*n* = 31)				
Weight (kg)	55.0 (7.8)	55.7 (8.6)	54.0 (6.8)	0.534
Height (cm)	164.1 (5.7)	163.1 (6.1)	165.5 (5.1)	0.245
BMI (kg.cm^−2^)	20.5 (2.8)	21.0 (3.1)	19.7 (2.3)	0.200
BMI *z*‐score	0.06 (1.02)	0.29 (1.1)	−0.26 (0.8)	0.122
Underweight *n*, (%)	2 (6.5%)	1 (7.7%)	1 (5.6%)	
Normal weight *n*, (%)	24 (77.4%)	13 (72.2%)	11 (84.6%)	
Overweight *n*, (%)	5 (16.1%)	4 (22.2%)	1 (7.7%)	
With obesity *n*, (%)	0	0	0	0.551

*Physical activity questionnaire* (*n* = 27)				
Organized PA before COVID (hours)	4.2 (4.9)	6.2 (5.6)	1.8 (2.2)	0.011
Organized PA at evaluation time (hours)	2.1 (4.0)	3.0 (5.0)	0.9 (1.8)	0.136
PA score	4.42 (2.1)	4.51 (1.4)	4.29 (2.9)	0.818

*Quality-of-life questionnaire* (*n* = 28)				
Physical functioning score	59.5 (25.7)	64.7 (21.1)	52.5 (30.3)	0.248
Emotional functioning score	61.4 (22.6)	64.8 (22.7)	56.8 (22.5)	0.359
Social functioning score	86.0 (15.8)	86.5 (14.6)	85.4 (18.2)	0.872
School functioning score	42.6 (30.7)	44.8 (33.2)	39.6 (28.2)	0.658
Overall quality of life score	62.4 (17.3)	65.2 (16.9)	58.6 (17.8)	0.330

*Questionnaires*				
Nijmegen score (*n* = 28)	26.39 (9.0)	24.8 (5.6)	28.6 (12.2)	0.328
FSS mean score (*n* = 26)	6.5 (1.6)	6.2 (1.9)	6.8 (1.0)	0.293
ADRS score (*n* = 25)	3.9 (2.5)	3.6 (2.3)	4.4 (2.8)	0.451

*Note:* PA score: minimal amount of PA performed per week (hour) at evaluation time.

Abbreviations: ADRS, adolescent depression rating scale; BMI, body mass index; COVID, Corona virus disease; FSS, fatigue severity scale; PA, physical activity.

### 3.2. Symptomatology

#### 3.2.1. Symptoms During Acute COVID‐19 Infection

All adolescents experienced symptoms during their acute COVID‐19 infection, including headache (17/31, 54.8%), anosmia or ageusia/dysgeusia (17, 54.8%), fatigue (16, 51.6%), fever (15, 48.4%), cough (15, 48.4%), odynodysphagia (10, 32.3%), rhinitis (9, 29%), muscle or joint pain (5, 16.1%), and digestive symptoms (5, 16.1%).

Additional symptoms reported included dyspnea, conjunctivitis, skin rash, dizziness, and paresthesia (11, 35.5%). Symptoms were mostly moderate (14, 45.2%) or mild (13, 41.9%) in intensity and lasted an average of 9.6 (±5.6) days. Only three subjects experienced severe symptoms, though none required hospitalization. Thirteen participants (38.7%) reported complete recovery before the onset of PCS′ symptoms.

#### 3.2.2. Symptoms of PCS

PCS symptoms are summarized in Table 2. Among all symptoms, exercise intolerance during light (14, 45.2%) or moderate (17, 54.8%) activity and severe fatigue were the most prominent and challenging, with 19 (61.3%) participants reducing or ceasing all PA. Symptoms were reported as fluctuating in 19 cases (61.3%).

**Table 2:** Post‐COVID syndrome symptoms.

	*N* (%)			*N* (%)
*General:* *n* = 25, 80.6%				
Severe fatigue	23 (74.2)		Hair loss	6 (19.4)
Sleep disorders	16 (51.6)		Skin rash	4 (12.9)
Muscular or joint pain	8 (25.8)		Fever	2 (6.5)
Fingers and toes complain	7 (22.6)		Weight loss	2 (6.5)

*Cardiorespiratory:* *n* = 31, 100%				
Dyspnea	26 (83.9)		Cough	5 (16.1)
Exercise intolerance	22 (71)		Palpitation	4 (12.9)
Chest pain	11 (35.5)		Nasal congestion	3 (9.7)

*Digestive:* *n* = 17, 54.8%				
Inappetence	12 (38.7)		Abdominal pain	8 (25.8)
Nausea	9 (29)		Diarrhea	1 (3.2)

*Neurological:* *n* = 27, 88.1%				
Headache	18 (58.1)		Anosmia	9 (29)
Orthostatism	17 (54.8)		Ageusia/dysgeusia	7 (22.6)
Cognitive dysfunction	16 (51.6)		Psychomotor slowing	5 (16.1)
Dizziness	12 (38.7)		Parosmia	3 (9.7)

*Psychological:* *n* = 20, 64.5%				
Scholar difficulties	13 (41.9)		Anxiety	11 (35.5)
Depression	12 (38.7)		Social withdrawal	6 (19.4)

Physical examinations were abnormal in four cases (12.9%), with findings including cutaneous papules in two patients, atrophic glossitis in one, and tachycardia with cold extremities in one. One participant had pathological Schellong test, demonstrating a systolic blood pressure drop of over 20 mmHg accompanied by loss of consciousness and three were diagnosed with postural orthostatic tachycardia syndrome (POTS). Blood test results were mostly normal; however, three participants had low levels of vitamin D and ferritin.

### 3.3. Questionnaires Results

Questionnaires res0ults are shown in Table 1. The overall QoL of the participants was comparable with that of children with major chronic conditions in 17 cases (55%). Two participants had scores comparable with those with moderate chronic conditions, and one to those with light chronic conditions. Only eight participants (26%) had normal QoL scores. All subjects had abnormal Nijmegen score (> 10); six (21.4%) exhibited mild hyperventilation symptoms, whereas 22 (71%) demonstrated significant dysfunctional breathing. The ADRS indicated that 11 adolescents were at low risk, 11 (35.5%) at moderate risk, and one at high risk for depression. All adolescents scored above the cutoff of 4 on the FSS, indicating positive results for fatigue.

Data on PA levels before the COVID‐19 infection were missing for four participants. Of the remaining 27, nine (33.3%) did not engage in any PA prior to the infection, whereas six (22.2%) (all girls) participated in more than the recommended 7 h per week.

### 3.4. Correlation Between Questionnaires and PCS Symptomatology

The total QoL score was negatively correlated to headache (*r* = −0.430, *p* = 0.022), fatigue score (*r* = −0.495, *p* = 0.016), and depression score (*r* = −0.703, *p* < 0.001). It was positively correlated with the PA score (*r* = 0.454, *p* = 0.017). Specifically, the mean PA score was higher for patients with a normal QoL score (5.8 (±1.9) versus 3.8 (±1.9); *p* = 0.027).

The physical functioning score was negatively correlated with fatigue (*r* = −0.512, *p* = 0.012) and Nijmegen scores (*r* = −0.444, *p* = 0.018). The emotional functioning score was negatively correlated with global neurological and psychologic symptoms (*p* = 0.037 and 0.013, respectively), as well as with orthostatic symptom, headache, and depression scores (*p* < 0.05 for all). The social functioning score was correlated with the PA score (*r* = 0.544, *p* = 0.003). The school functioning score was negatively correlated with the depression score (*p* < 0.05 for all). The presence of symptoms during CPET was negatively correlated with the physical functioning score (*r* = −0.447, *p* = 0.017) and positively correlated with the Nijmegen score (*r* = 0.441, *p* = 0.019).

Subjects at risk for depression had a lower QoL score (50.3 (±8.2) versus 75.3 (±8.7), *p* < 0.001) with a higher prevalence of headache (*p* = 0.003).

### 3.5. CPET Results

The CPET was performed 9.1 (±4.8) months (range: 3–20) after acute COVID‐19 infection. A maximal test was reached in 27 (87%) subjects. The mean Borg scale was 7.8 (±1.1) (range: 6–9). Seven (22.6%) showed an abnormal O_2_ pulse zs, the FEV_1_ zs was reduced in two subjects, FVC zs in 3, FEV_1_/FVC zs in 4, BR was below 20% in 2, breathing efficiency (VE/VCO_2_ slope) was above 35 in 4, and partial oxygen saturation (SpO_2_) was below 94% in 2. The reasons for test termination were muscular pain in 12 (38.7%), dyspnea in 9 (29%), fatigue in 2 (6.5%), and a combination of dyspnea and muscular pain in 7 (22.6%). There were no rhythm disturbances beyond those present at rest (right bundle branch block in 4 and Wolf–Parkinson–White syndrome in one). During the recovery phase, HR decreased by 27.2 (±8.4) beats in the first minute (range: 11–42). Exercise‐associated dysautonomia was present in 9/29 (31%) of the adolescents, and 15 (51.7%) met 2 out of 3 of the criteria. All patients who had a pathological Schellong test or POTS presented at least two criteria for an exercise‐associated dysautonomia.

Eleven (35.5%) subjects experienced one or more symptoms at maximal exercise or during recovery: excessive dyspnea or stridor in 8 (25.8%), dizziness in 4 (12.9%), chest pain in 4 (12.9%), symptomatic hypotension in 3 (9.7%), malaise in 2 (6.5%), and nausea in 1. There was no association between exercise‐associated dysautonomia and symptoms during the test.

Thirteen (42%) adolescents had a low peakVO_2_ zs. There were no differences between normal and low CRF groups for all the parameters presented above, including for exercise‐associated dysautonomia, except for FVC and O_2_ pulse zs, which were significantly more often abnormal in the low CRF group (abnormal O_2_ pulse zs in 6/13 [46.2%] vs. 1/17 [5.9%], *p* = 0.10; abnormal FVC zs in 3/13 [23.1%] vs. 0/18, *p* = 0.032). CPET results showed that test duration, FEV_1_ and FVC zs at rest, peak HR, O_2_ pulse zs, and the BR were lower in the low CRF group despite achieving maximal test criteria (*p* < 0.05 for all). Since the number of hours of organized PA before COVID‐19 infection differed between groups (Table 1), we adjusted our analysis for this variable and found that only O_2_ pulse zs remained significantly different between groups.

Resting spirometry and CPET results for the whole cohort and according to CRF groups are presented in Table 3.

**Table 3:** CPET results.

	Whole cohort *N* = 31	Normal CRF *N* = 18	Low CRF *N* = 13	
	**Mean (**SD)			*p*
Hemoglobin (g/dL)	13.9 (1.3)	13.8 (1.2)	14.0 (1.4)	0.766
Test duration (minutes)	9.4 (1.5)	10.0 (1.3)	8.5 (1.2)	0.003

*Spirometry at rest before CPET test*				
FEV_1_ *z*‐score	0.08 (1.2)	0.55 (1.1)	−0.58 (1.0)	0.006
FVC *z*‐score	0.23 (1.9)	0.85 (2.1)	−0.62 (1.1)	0.020
FEV_1_/FVC ratio *z*‐score	0.19 (1.4)	0.03 (1.3)	0.41 (1.5)	0.478

*CPET variables at peak exercise*				
PeakVO_2_ (mL/kg/min)	30.5 (6.5)	33.2 (6.6)	26.8 (4.5)	< 0.001
PeakVO_2_ *z*‐score	−1.1 (1.2)	−0.32 (0.8)	−2.2 (0.5)	0.003
Workload (*W*)	127.1 (27.8)	140.1 (23.8)	109.2 (23.0)	0.001
Workload *z*‐score	−1.2 (1.1)	−0.47 (0.8)	−2.1 (0.5)	< 0.001
Peak HR (beat/min)	174.2 (13.9)	178.8 (11.3)	167.8 (15.0)	0.036
% of predicted max HR (220‐age)	91.7 (7.4)	94.3 (6.1)	88.2 (7.9)	0.029
O_2_ pulse (mL/beat)	9.4 (1.8)	9.9 (1.7)	8.6 (1.6)	0.037
O_2_ pulse *z*‐score	−0.80 (0.9)	−0.31 (0.8)	−1.46 (0.6)	< 0.001
RR (per min)	42.1 (9.4)	42.8 (9.1)	41.2 (10.0)	0.674
BR (%)	40.7 (12.0)	36.9 (11.0)	45.9 (11.9)	0.044
VE/VCO_2_ slope	30.7 (4.2)	30.8 (4.9)	30.7 (3.0)	0.954
VE/VO_2_	34.6 (5.8)	34.8 (6.4)	34.3 (5.2)	0.789
V_D_/V_T_	0.17 (0.07)	0.15 (0.05)	0.20 (0.08)	0.093
SpO_2_ (%)	96.0 (1.5)	96.4 (1.4)	95.5 (1.6)	0.103
PaO_2_ mmHg	90.0 (9.5)	90.3 (7.2)	89.5 (12.8)	0.860
P(A‐a)O_2_ mmHg	25.1 (8.6)	25.7 (7.2)	24.1 (10.9)	0.676
P_ET_CO_2_ (mmHg)	35.4 (4.5)	35.3 (4.5)	35.5 (4.5)	0.939
RER	1.13 (0.07)	1.13 (0.08)	1.12 (0.04)	0.499
pH	7.32 (0.03)	7.32 (0.03)	7.32 (0.03)	1.0
Lactate (mmol/L)	7.9 (1.8)	8.2 (1.8)	7.3 (1.6)	0.159

*CPET variables at VT1*				
VO_2_ at VT1 (mL/kg/min)	17.8 (4.9)	18.8 (5.2)	16.3 (4.2)	0.152
VO_2_ at VT1 (% of predicted maxVO_2_)	42.0 (10.7)	44.8 (11.5)	37.9 (8.1)	0.066

*Exercise-associated dysautonomia*				
• Resting HR > 75 bpm, *n* (%)	29 (93.5)	17 (94.4)	12 (92.3)	0.811
• A difference between peak HR and resting HR < 89 bpm, *n* (%)	21 (67.7)	10 (55.6)	11 (84.6)	0.088
• HR recovery in the first minute < 25 bpm, *n* (%)	14 (48.3)	8 (50)	6 (46.2)	0.837
Meeting two criteria, *n* (%)	15 (51.7)	7 (43.8)	8 (61.5)	
Meeting three criteria, *n* (%)	9 (31)	5 (31.3)	4 (30.8)	0.978

Abbreviations: BMI, body mass index; BR, breathing reserve; CPET, cardiopulmonary exercise test; CRF, cardiorespiratory fitness; FEV_1_, forced expiratory volume in 1 s; FVC, forced vital capacity; HR, heart rate; O_2_ pulse, oxygen pulse; PaO_2_, partial pressure of oxygen; P(A‐a)O_2_, alveolar‐arterial gradient; P_ET_CO_2_, partial pressure of end‐tidal CO_2_; RER, respiratory exchange ratio; RR, respiratory rate; SpO2, partial oxygen saturation; V_D_/V_T_, physiologic dead space over tidal volume ratio; VE/VCO_2_, minute ventilation/carbon dioxide production; VO_2_, oxygen consumption; VT, ventilatory threshold.

When comparing symptomatology between normal or low CRF groups, we found no difference in acute COVID‐19 infection presentation or in the number of PCS symptoms in each category presented in Table 2 (*p* > 0.05 for all). However, orthostatic symptoms were more frequently present in the low CRF group (10/13 [76.9%] vs. 7/18 [38.9%], *p* = 0.036), and all subjects reporting hair loss were in the low CRF group (*p* = 0.001). We observed that the intensity of activity at which dyspnea was experienced, as well as physical and blood exam results, did not differ between CRF groups.

There was no difference in QoL or other questionnaire results between subjects with normal or low CRF. Regarding PA, the normal CRF group reported significantly more PA before COVID‐19 infection than the low CRF group (no PA before 2/15 [13.3%] vs. 7/12 [58.3%], *p* = 0.014), and the number of hours performed was also significantly higher (Table 1). However, the number of hours of organized PA or the PA score at the time of evaluation was similar between both groups.

### 3.6. PFTs Results

The PFT was performed an average of 14.4 (±27.2) days before or after the CPET (range: 0–77 days). No subjects showed signs of obstructive or restrictive airflow diseases, as FEV_1_, FVC, and FEV_1_/FVC ratio zs were within normal ranges for all participants. Only one adolescent had a reduced TLC zs, although his FVC and FEV_1_/FVC ratio zs were normal. Two subjects (8%) showed gas transfer impairment (D_LCO_zs < −1.64), 14 (45.2%) met the criteria for static air trapping, and none presented dynamic air trapping. Two participants had a positive response to bronchodilator medication.

When comparing subjects with normal and low CRF, we found that RV, FRC (%), and RV/TLC predicted ratio (%) were significantly higher in the low CRF group, with a greater number of subjects meeting the criteria for static air trapping (9/12 [75%] vs. 5/15 [33.3%], *p* = 0.031) in the low CRF group. Since the number of hours of organized PA before COVID‐19 infection differed between groups, we adjusted our analysis for this variable and found that only the RV/TLC predicted ratio (%) remained significantly different between groups. The results of the PFT are presented in Table 4.

**Table 4:** Pulmonary Function Test results.

	Whole cohort *N* = 31	Normal CRF *N* = 18	Low CRF *N* = 13	
	**Mean (**SD)			*p*
*Mechanics*				
FVC *z*‐score	−0.008 (0.88)	0.26 (0.86)	−0.36 (0.81)	0.063
FEV_1_ *z*‐score	0.26 (0.77)	0.02 (0.88)	0.26 (0.86)	0.174
FEV_1_/FVC ratio *z*‐score	0.52 (1.06)	0.36 (1.05)	0.74 (1.09)	0.364

*Volumes*				
VC (liter)	3.4 (0.5)	3.5 (0.5)	3.4 (0.6)	0.628
RV (liter)	1.7 (0.7)	1.5 (0.4)	2.1 (0.8)	0.027
TLC *z*‐score	0.85 (1.24)	0.76 (1.12)	0.97 (1.42)	0.674
FRC (%)	123.6 (35.4)	110.9 (26.3)	139.6 (39.7)	0.045
RV/TLC predicted ratio (%)	142.6 (40.9)	122.3 (26.7)	168.0 (42.2)	0.004
FVC/VC (liter)	106.5 (7.8)	104.2 (9.0)	109.3 (5.1)	0.075

*Diffusion*				
D_LCO_ *z*‐score	0.93 (3.2)	1.34 (4.4)	0.52 (1.2)	0.549
K_CO_ corrected (DLCO/Liter)	5.2 (1.0)	5.3 (1.4)	5.1 (0.5)	0.606
K_CO_ *z*‐score	0.58 (1.31)	0.68 (1.72)	0.49 (0.80)	0.741
V_A_ (liter)	4.6 (0.8)	4.4 (0.8)	4.8 (0.7)	0.247

Abbreviations: D_LCO_, diffusing capacity of the lung for carbon monoxide; FEV_1_, forced expiratory volume in 1 second; FVC, forced vital capacity, FRC: functional residual capacity; K_CO_, carbon monoxide transfer coefficient; RV, residual volume; TLC, total lung capacity; VA, alveolar volume; VC, vital capacity.

PFT parameters showed associations between RV/TLC predicted ratio, FVC zs, or FEV1 zs and peak workload zs (*p* < 0.05 for all); and the FRC ratio was correlated with BR (*r* = 0.458, *p* = 0.06). Additionally, the O_2_ pulse zs was lower in subjects with static air trapping (−1.35 [±0.76] vs. −0.52 [±0.87], *p* = 0.017). We found no association between peak VO_2_ zs or VO_2_ at VT1 and PFT parameters.

## 4. Discussion

This study is aimed at describing the clinical presentation of children and adolescents with dyspnea in the context of PCS, who were referred for CRF evaluation, and to identify factors distinguishing young people with normal and abnormal CRF. We also sought to characterize the relationship between symptomatology and psychological and physical aspects.

The prevalence of symptoms reported by our patients is significantly higher compared with the literature on PCS in young people [1, 30]. This discrepancy may be attributed to our cohort being specifically referred for exercise intolerance or dyspnea, which may indicate a more severe presentation of the disease. Consequently, our sample may not represent the general adolescent′s population with PCS [31].

All participants presented hyperventilation symptoms and severe fatigue according to the Nijmegen and FSS questionnaires, with 35% at moderate risk for depression. This prevalence is notably higher than expected in a healthy adolescent population. A recent meta‐analysis of 15 European studies reported a 16% prevalence of depression among European adolescents [32].

Three‐quarters of our adolescents also showed a QoL reduction comparable with that of individuals with major chronic conditions. Headaches, fatigue, and depression were the primary symptoms impacting QoL scores. Previous studies also reported reduced QoL among children and adults with PCS [33, 34], which negatively impacts functional status [35]. We observed a strong relationship between QoL and PA: the less active the participants, the lower their QoL scores, with 60% of our subjects having decreased or ceased their usual physical activities. However, the causal link between these two factors is complex and likely bidirectional. PA has been shown to positively affect QoL [36], but the symptoms that reduce QoL in our patients may also limit their ability to engage in PA.

Nearly half of our subjects had low CRF despite reaching maximal test thresholds. To date, only one other study has investigated CRF in 20 children and adolescents with PCS, comparing them with a cohort with COVID‐19 but without PCS criteria [7]. It found that CRF was lower among PCS participants, especially females, which was attributed to peripheral deconditioning.

The origin of reduced physical capacity in PCS appears to be multifactorial [37]. We examined various factors potentially related to this disparity and found no significant differences in acute infection presentation, disease duration, or PCS symptomatology, except for orthostatism, which was nearly twice as common in the low CRF group. Notably, the adolescents reported severe fatigue, exertional dyspnea, hyperventilation, and depression at similar levels regardless of CRF outcomes. The fact that more physically active participants before COVID‐19 reported exertional dyspnea despite normal CRF suggests they may have experienced a decrease in VO_2_ with subjective limitations relative to their pre‐COVID fitness. However, without pre‐COVID CPET data, we cannot assess changes in VO_2_. Two studies in military recruits and professional athletes, however, have reported reduced CRF postinfection [38, 39], implying a similar trajectory for our previously active patients. This difference between low and normal CRF groups may therefore reflect deconditioning rather than a physiological consequence of COVID‐19 infection. Interestingly, VO_2_ at VT1, often indicative of deconditioning [40], was similar between the two groups, mirroring findings in other pediatric studies comparing PCS subjects with controls [7].

Furthermore, studies in adults with PCS have shown that deconditioning often follows hospitalization [5], which was not the case for any of our patients. In our study, both groups engaged in similar levels of PA at the time of the CPET evaluation when measured according to intensity (PA score), rather than simply by hours of organized PA. Therefore, other explanations, such as dysfunctional breathing [41], chronotropic incompetence, or abnormal peripheral oxygen extraction, must be explored to account for this difference in physical capacity. This aligns with conclusions from other studies conducted in adults, as reported in the meta‐analysis by Durstendfeld et al. [5].

Most of our subjects had normal ventilatory parameters, with only three showing obstructive signs on spirometry. However, we found that static air trapping, reflecting pulmonary hyperinflation, was more frequent in the low CRF group. This could indicate early signs of airway obstruction or respiratory muscle weakness. Pulmonary hyperinflation is known to increase the work of breathing and lead to diaphragmatic dysfunction and impaired ventilation [42]. Studies in adults have also reported air trapping on CT scans of COVID‐19 survivors [43, 44]. In our study, participants with static air trapping had lower O_2_ pulse scores. Since O_2_ pulse depends on ejection fraction and arteriovenous oxygen difference [45], a reduced O_2_ pulse may suggest cardiac dysfunction, reduced peripheral diffusion capacity, or lower arterial oxygenation due to pulmonary limitations. The latter could explain the impact on CRF, as seen in invasive CPET studies in adults [6]. Moreover, a recent pilot study in children with PCS revealed lung perfusion defects without morphological alterations in almost 43% of participants, possibly linked to chronic endothelial dysfunction [46]. It is plausible that reduced CRF in our patients may result from impaired oxygen extraction. Indeed, muscle pain rather than dyspnea was the main exercise‐limiting symptom reported, a clinical manifestation of peripheral limitations. Similar findings have been reported in children [7] and in adults, where endothelial dysfunction and muscle or mitochondrial abnormalities have been implicated [47, 48].

PCS has also been associated with autonomic dysfunction [49, 50]. Dysautonomia, characterized by dysregulated autonomic functions like blood pressure and HR, was present in 31% of our participants during exercise, based on Jouven′s criteria, with three also experiencing symptomatic hypotension. Compared with a study of PCS in adults [51], our adolescent population appeared to show a lower prevalence of chronotropic insufficiency (defined as a peak—resting HR difference < 89 bpm), affecting approximately 66% of adolescents versus nearly 82% of adults. In contrast, abnormal HR recovery was more frequent in adolescents (48% vs. 33%). However, because normal reference values differ substantially between pediatric and adult populations, it is difficult to draw firm conclusions about whether these findings reflect a truly different physiological response. All the patients who showed abnormal Schellong test at rest were also presenting dysautonomia during exercise. Symptoms possibly linked to dysautonomia, such as excessive dyspnea, dizziness, chest pain, malaise, and nausea, were noted in 35% of participants during recovery. This prevalence is higher than expected in a typical pediatric population. Internal data from our center found that 19% of adolescents without cardiopathy reported such symptoms during the CPET. Furthermore, the high Nijmegen scores among our PCS subjects suggest that hyperventilation and dysfunctional breathing could stem from autonomic dysfunction, which was associated with lower physical functioning scores, affecting daily life. Although adult studies suggest a link between dysautonomia and reduced CRF [52], we did not observe this association, as symptoms and test results were similar across CRF groups.

It is worth noting that the majority of our CPET referrals were female. This observation is surprising, as adolescent girls generally engage in less PA than boys [53] and might be expected to be less impacted by exertional intolerance in daily life. Gender differences in PCS prevalence have also been reported in adults [54], and several hypotheses have been proposed, such as hormonal differences or IgG antibodies production leading to heightened hyperinflammatory response during acute COVID‐19 [55]. Additionally, studies have shown abnormal diffusion capacity and exercise intolerance to be more frequent in females with PCS [56]. However, due to the low number of male participants, we could not compare genders for these factors in our study, where 90% of the subjects were female.

This study, while providing insights into the clinical and physiological profile of children and adolescents with PCS, has several limitations that may impact the generalizability and interpretability of our findings. The limited sample size in our study may reduce the ability to generalize findings to the broader population of adolescents with PCS. Furthermore, our cohort was specifically referred for evaluation due to complaints of exercise intolerance or dyspnea, potentially introducing referral bias. As a result, this subset may reflect a more severe presentation of PCS, and thus, may not fully represent the entire spectrum of adolescents affected by the condition, including those with milder or alternative symptom profiles. A major limitation of our study is the absence of pre–COVID‐19 baseline data on participants′ CRF, psychological status, and QoL. Without this baseline information, it is difficult to quantify the degree of decline in CRF or to attribute changes in psychological and QoL metrics directly to COVID‐19. The absence of a matched control group of adolescents who recovered from COVID‐19 without developing PCS symptoms limits the ability to differentiate the specific effects of PCS from the general effects of post‐COVID recovery. Addressing these limitations in future studies could provide a more comprehensive understanding of CRF and associated symptomatology in adolescents with PCS, leading to improved management strategies for this population.

Finally, it should be emphasized that the comparisons with adult data are limited because comorbidities, cutoffs, and reference values differ between pediatric and adult populations, and any conclusions drawn from these contrasts should therefore be interpreted with caution.

## 5. Conclusions

Our findings highlight the complex and multifactorial nature of exercise intolerance in adolescents with PCS. Beyond the high prevalence of symptoms such as fatigue, hyperventilation, and reduced QoL, our study reveals that reduced CRF is not solely explained by physical inactivity or deconditioning. Instead, potential contributors such as pulmonary hyperinflation, impaired oxygen extraction, and autonomic dysfunction may play a significant role. These insights suggest that PCS in adolescents may involve subtle physiological alterations that are not captured by standard clinical assessments. By stratifying patients based on CRF, we were able to uncover nuanced differences that could inform more targeted diagnostic and therapeutic approaches. Thus, our study addresses the initial need to better understand the mechanisms underlying exercise intolerance in PCS and underscores the importance of comprehensive physiological evaluation in this population.

### 5.1. Strengths of This Study

• Larger study exploring CRF with CPET, as well as pulmonary function testing in children and adolescents with PCS.
• Exploration of many health‐related parameters that can be affected by PCS in a single pediatric study.

### 5.2. Limitation of This Study

• Absence of pre–COVID‐19 baseline data.
• The limited sample size reduces the ability to generalize the findings.
• The predominance of girls limits the gender comparison.

**Nomenclature**
BMI: body mass index
BR: breathing reserve
COVID: corona virus disease
CPET: cardiopulmonary exercise test
CRF: cardiorespiratory fitness
FEV_1_
: forced expiratory volume in 1 s
FVC: forced vital capacity
HR: heart rate
O_2_ pulse: oxygen pulse
PaO_2_
: partial pressure of oxygen
P(A‐a)O_2_
: alveolar‐arterial gradient
P_ET_CO_2_
: partial pressure of end‐tidal CO_2_
PFT: pulmonary function test
RER: respiratory exchange ratio
RR: respiratory rate
RV: residual volume
PCS: post‐COVID syndrome
POTS: postural orthostatic tachycardia syndrome
SpO_2_
: partial oxygen saturation
TLC: total lung capacity
VC: vital capacity
VCO_2_
: carbon dioxide elimination
V_D_/V_T_
: physiologic dead space over tidal volume ratio
VE: minute ventilation
VE/VCO_2_
: breathing efficiency
VO_2_
: oxygen consumption
VT1: ventilatory threshold 1
W: watt
ZS: z‐score

## Author Contributions

Albane Bertha Rosa Maggio conceptualized and coordinated the study, collected and analyzed the data, performed the statistical analysis, and drafted the initial manuscript. Ivan Perret, Anne Perrin, and Nuseibah Alramadina collected and analyzed the data. Constance Barazzone participated in the study design. Anne Mornand analyzed the data and drafted the initial manuscript.

## Funding

No funding was received for this manuscript. Open access publishing facilitated by Universite de Geneve, as part of the Wiley ‐ Universite de Geneve agreement via the Consortium Of Swiss Academic Libraries.

## Disclosure

All authors approved the final manuscript as submitted. No other persons or third‐party services were involved in the research or manuscript preparation.

## Conflicts of Interest

The authors declare no conflicts of interest.

## Data Availability

The data that support the findings of this study are available from the corresponding author upon reasonable request.
